# The Surface Conditions and Composition of Titanium Alloys in Implantology: A Comparative Study of Dental Implants of Different Brands

**DOI:** 10.3390/ma15031018

**Published:** 2022-01-28

**Authors:** Alex Pérez Tchinda, Gaël Pierson, Richard Kouitat-Njiwa, Pierre Bravetti

**Affiliations:** Institut Jean Lamour, UMR 7198, Université de Lorraine, CEDEX, 54011 Nancy, France; gael.pierson@univ-lorraine.fr (G.P.); richard.kouitat@univ-lorraine.fr (R.K.-N.); pierre.bravetti@univ-lorraine.fr (P.B.)

**Keywords:** titanium, implants, surface, sandblasting, composition

## Abstract

The success of titanium dental implants depends on their osseointegration into the bone, which is determined by the composition and surface properties of the implant in close contact with the bone. There is a wide variety of implants on the market. Is it possible to identify the implant with the best composition and surface topography for optimal osseointegration? To this aim, 13 brands of dental implants from nine distinct manufacturers have been selected and their composition and surface topography determined. The obtained results show differences between these implants, in this case, the Ssk averages of the three measurements performed on each implant were positive, or 0.4 (0.1–0.8), indicating that the roughness of all implants analyzed was primarily textured and not flat. Like Sa, we found the highest Sdr for implants subjected only to sandblasting. In addition, only the ALS-active^®^ implant had a modified microstructure on its surface. However, analysis of the NANOTITE implant surface revealed a 1.40% presence of calcium which we consider too low to have an effect on bone formation around the implant. As a result, we have also highlighted the lack of a recognized independent standard for dental implant surface conditions and the lack of independent quality control vis-à-vis manufacturers. Of all the surface types studied, none proved more satisfactory than another.

## 1. Introduction

Per-Ingvar Brånemark [1], proved the long-term success of titanium implants and laid the foundations of modern implantology. Titanium-based implant systems, though considered as the gold standard for rehabilitation of edentulous spaces, have been criticized for many inherent flaws.

Bone healing around dental implants follows the pattern and sequence of intramembranous osteogenesis with the formation of woven bone first of all followed later by the formation of parallel-fibered and lamellar bone. Bone apposition onto the implant surface starts earlier in trabecular bone than in compact bone. While the first new bone may be found on the implant surface around one week after installation, bone remodeling starts at between 6 and 12 weeks and continues throughout life. Bone remodeling also involves the bone–implant interface, thus transiently exposing portions of the implant surface [2].

In vitro studies analyzed cultures of pure titanium particles suspended with cells [3]. In weak concentrations, the titanium did not interfere with either the morphology or proliferation of bovine fibroblasts, while high concentrations of titanium were toxic and caused loss of proteolytic and collagenolytic activity [3].

Furthermore, in vivo studies reported metallic particles appearing due to friction in joint prostheses and implants with the possibility of movement [4,5,6]. These particles can cause harmful reactions in peri-implant tissues, such as osteolysis, bone structure deterioration, the appearance of fibrous capsules, and chronic inflammation. All these conditions can lead to the prosthesis or implant becoming unstable or completely loose [4,5,6]. The size of these particles and their composition are the primary factors in how harmful they can be. They have only been reported in cases of Ti-6Al-4V prostheses, however [6,7] and not with pure titanium implants.

Titanium is a highly reactive metal, relatively unstable compared to its oxide, reacting both to air and water, and metallic dental implants acquire a surface layer of oxidation near-instantaneously in room temperature with the presence of air or water. Yet it is this very reactivity that, paradoxically, lends the metal its high resistance to attack from aqueous environments. Titanium is, in fact, not a very noble metal itself (−1.75 V potential), yet it is protected by the layer of titanium oxide that forms spontaneously, and reforms rapidly after being pierced. Due to this, titanium is nowadays considered the most resistant metal to corrosion. In the air, at room temperature, an oxidation layer of up to 1.7 nm thickness forms after two days, and up to 3.5 nm after 40 days [8,9]. It is important to note, however, that the oxide layer does not solely consist of titanium dioxide (TiO_2_), but also contains several other oxides: TiO, TiO_2_, Ti_2_O_3_, Ti_3_O_4_ [8,9]. Due to this passivation, titanium is more resistant to corrosion than most other metals used in medicine.

However, Peri-implantitis is defined as an inflammatory process affecting all the tissues around an implant resulting in bone destruction. In parallel, the lesion of the mucous membrane is accompanied by a deep pocket with bleeding and/or suppuration and loss of marginal bone corresponding to a defect in the maintenance of osteoporosis. In most cases, peri-implant bone is radiologically lysed in the shape of a crater or a cell death ring around the implant [10]. These peri-Implantar pathologies (mucosity and peri-implantitis) are inflammatory diseases of infectious origin manifesting themselves in a progressive alteration of the bone anchorage system and the peri-implantar soft tissues. According to its parameters, peri-implantitis prevalences are found in 28–56% of subjects and in 12–43% of implants [11]. A study conducted with my team showed that anaerobic bacteria of the genus *Desulfovibrio fairfieldensis* are able to create colonies of biofilms on titanium coupons and proliferate in vitro under oral physiological conditions [12].

On the other hand, a study suggests that rough implant surfaces due to the presence of microroughness; irregularities and crevices provide better cellular anchorage, but also suitable shelters to bacterial colonies that shelter there to escape the shear forces of the oral environment and by ricochet, promotes the development of biofilm [13].

For this, understanding the mechanisms at play at the implant-tissue interface is the primary focus of many researchers working on improving even further the quality of implant treatments, especially related to the surfaces of implants. The optimal choice of material for implants is thus far from defined as of today, as is the optimal surface condition.

In Section 2 below, the material and the adopted methods of analysis are presented. Section 3 is devoted to the obtained results. A discussion of these results is carried up in Section 4. The paper ends with a conclusion and perspectives in Section 5.

## 2. Material and Methods

The global characteristics of the 13 implants considered in this work are gathered in Table 1 below. This shows the manufacturer, and specifies the size of the implant as well as its classification.

### 2.1. Surface Roughness

We sought to characterize the surface roughness of 13 different implants from nine different manufacturers (Table 1). The quality of the implant surfaces studied was, in all cases, produced by subtractive manufacturing processes, yet three different groups can be distinguished within this family:

Group A: Acid-etched-only implant (n = 1):

Osseotite^®^ (Zimmer Biomet, Warsaw, IN, USA) 4 mm diameter and 13 mm length implant with a double acid-etched surface (products not communicated).

Group B: Sand-blasted-only implants (n = 2):

The Evolution Implant^®^ (Serf, Décines, France), 4 mm in diameter by 14 mm in length, sandblasted with alumina particles.

The Leone^®^ Implant (Leone, Firenze, Italy), 4.1 mm in diameter by 12 mm in length, sandblasted only (sandblasting product not communicated).

Group C: Sandblasted then acid-etched implants (n = 9):

SLA^®^ and SLA-active^®^ implants (Straumann, Basel, Switzerland), 4.1 mm in diameter by 14 mm in length, sandblasted with corundum and etched with hydrochloric and sulfuric acid.

Id^all®^ and Idcams^®^ implants (Implant Diffusion International, Montreuil, France), 4 mm in diameter by 11 mm in length, sandblasted (product not communicated) and acid-etched (product not communicated).

The Osseospeed^®^ Implant by Astratech dental (Astratech, Charlotte, NC, USA), 4 mm in diameter by 17 mm in length, sandblasted with titanium oxide beads and etched with hydrofluoric acid.

The Natea^®^, Universal and Natural implants (Euroteknika, Sallanches, France), 4.8 mm in diameter by 14mm in length, sandblasted with titanium oxide beads and etched with hydrofluoric acid.

The In-Kone-Universal Implant^®^ (Tekka, Québec, Canada), 4.5 mm in diameter and 10 mm in length, sandblasted with corundum and acid etched (product not communicated).

The Axone^®^ Implant (Anthogyr group, Straumann, Basel, Switzerland), 4 mm in diameter and 11mm in length, sandblasted with Biphasic Calcium Phospate (BCP) and acid-etched (product not communicated).

### 2.2. Characterization of Surface Roughness

- Confocal Microscopy

Confocal microscopy is a non-destructive technique enabling optical slices to be obtained not only in (X,Y) planes but also in (X,Z) planes parallel to the optical axis, which can then be used to build three-dimensional reconstructions. These “virtual” optical slices have no effect on the sample’s integrity, unlike physical slices used in electronic microscopy.

The images were acquired using a Zeiss LSM 700 confocal laser-scanning microscope with a 405 nm wavelength and 20× objective, fitted with a 0.75 numerical aperture (Zeiss Plan-Apochromat, Zeiss, Oberkochen, Germany). This objective covers an area of 200 µm × 200 µm. We opted to use a 512 × 512 pixel matrix, giving a lateral resolution of 0.4 µm. The optical section thickness was 4 µm. The emitted light was collected using a high-pass filter. The chosen cut-off point was 10 µm.

In order to record roughness values representative of the entire dental implant, measurements were taken at the head, in the middle and at the apex [14].

### 2.3. Roughness Parameters

Choosing parameters for roughness is difficult since there are so many possibilities and their significance is sometimes vague, as well as there being no universal parameter of roughness enabling all surfaces to be characterized in a pertinent way. Each roughness parameter indicates a different specific characteristic of the surface under analysis. Seven amplitude parameters and one hybrid parameter, which together should, according to the literature, enable a description of the implant’s surface roughness were chosen [14]. All these parameters are given terms beginning with “S” as the measures were all taken in three dimensions.

### 2.4. Microstructural Characterization

Analysis of microstructures was performed on the three following samples chosen from the 12 implants: Osseotite^®^, Osseospeed^®^ and SLA active^®^. These three implants, according to the manufacturer’s information, were subjected to different surface processes during manufacturing.

- Sample Preparation

The first step of preparing the samples consisted of preparing transversal then longitudinal sections of each implant using a microsaw. This step enabled us to avoid the implant’s screw indentations and to obtain thin implant slices only a few millimeters thick.

The implant slices were then coated with an auto-polymerized resin to facilitate polishing. The following preparation step was to polish the samples in order to obtain a mirror-like polished surface for observation under scanning electron microscope. To achieve this, several different types of sandpaper (Escil^®^, Chassieu, France) were used, of increasing grain size (600, 800, 1200, 2400, 4000), under running water, and the final step was an oxide polishing suspension OPS (Struers^®^, Ballerup, Danemark). The samples were then placed in an ultrasound bath for cleaning. In order to be able to clearly observe the samples’ microstructure, all were subjected to chemical attack using Kroll’s reagent (HF/HNO_3_ mixture, equivalent to 5% in water).

B. Scanning Electron Microscope Image Acquisition

The images were acquired using a scanning electron microscope with field-emission cathode (Philips XL30 SFEG) (Philips, Eindhoven, The Netherlands) in secondary electron mode and accelerating voltage of 5 kV. Energy dispersive spectroscopy (EDS) was used for semi-quantitative analysis of the chemical elements, recorded with an acquisition time of approximately 100 s. The data collected were analyzed initially by identifying the X-ray spectrum using the software provided with the EDS system. To achieve this, the software compares the kinetic energy signatures (K_u1_, K_u2,_ …) of the X-rays characterizing each element, obtained experimentally, with those of the reference table generated by analysis of standard samples. The quantification is then calculated by integrating these X-ray energy signatures obtained. ZAF corrections must still then be performed, however, in order to obtain a semi-quantitative analysis of the elements contained in each phase.

### 2.5. Quantitative Characterization of the Phase Rates

The phase fractions were determined by image analysis. This analysis was performed using 20 SEM micrographs, taken at random in back-scattered electron mode using the APHELION image processing software (Saint Contest, France). This program performs several operations on the original images. First, a low-pass filter was applied to eliminate a maximum of background noise, then thresholding was performed to obtain binary images. Finally, the images underwent erosion and reconstruction to eliminate any unwanted small objects remaining following thresholding. Once the binary image was generated, we could determine the surface phase fractions. The phase fractions we analyzed were the averages calculated from the results generated by the 20 images.

### 2.6. Implant Composition

The different implants were characterized according to alloy and surface treatment used. We used the field-emission cathode SEM Hitachi S-4800, which is optimized for high resolution (from micrometer to nanometer). This device uses an electron beam to enable observation of fragile materials such as polymers, semi-conductors or typically any material or structure associated with nanotechnology.

The implants’ compositions were studied using EDS with electronic microscopy. EDS is a technique used to determine the composition of a sample or specific area of a sample.

## 3. Results

### 3.1. Surface Roughness

- Sa, Sq

The processes used on the surfaces of all the studied implants can be categorized as the same type: subtractive.

We first considered the Sa and Sq parameters, which both quantify the overall height of peaks and valleys of the surface under analysis, although Sq is the more sensitive parameter. We noted that the Sa and Sq values varied significantly depending on the implant surface being analyzed. The In-Kone-Universal surface presented the smallest Sa value (1.7 µm, range: 1.7–1.8) and a Sq of 2.3 µm (2.1–2.4) (Table 2). The highest Sa and Sq values, for comparison, were 3.7 µm (3.4–4) and 4.9 µm (4–5.3), respectively, for the SLA active. We observed that these two implants, from different manufacturers, generated very different results despite undergoing the same processes. We can therefore now pose the question of what effect the microstructure of an implant has on its degree of roughness.

The mean Sa and Sq values for the 13 implants studied were 2.7 µm (1.7–1.7) and 3.5 µm (2.3–4.9), respectively, which is entirely in line with the literature. Bila et al., for example, reported in 2003 Sa values of 2–3.5 µm for implants manufactured using subtractive treatments. Though all the processes were subtractive, there are still significant differences. Hence, as mentioned above, our decision to divide the implants into three different categories. We then proceeded to combine the Sa and Sq parameters of these three categories. In Group A (n = 1), the mean Sa was 2.2 µm (2.1–2.4) and mean Sq was 2.9 µm (3.2–3.3). For Group B (n = 2) it was 2.9 µm (2.4–3.7) and 3.8 µm (3.1–4.9), and, finally, for Group C the means were Sa = 2.6 µm (1.5–4.7) and Sq = 3.8 µm (1.9–6.3). The greatest roughness values were found in Group B, which comprised implants that had only undergone sandblasting. As was expected, implant surfaces that had only been acid etched presented lower roughness values.

B. Sku

This excessive flatness (excess kurtosis) is a source of some ambiguity. Under this new definition, a positive excess kurtosis corresponds to a distribution of peaks and negative excess kurtosis to flat distribution. As for implant roughness, the higher the Sku value, the more pointed the peaks, and the closer they are grouped. The mean Sku for the implants we tested was 4.6 (2.9–11.1), with a maximum mean of 8.9 (7.2–11.1) produced by the Osseospeed implant. The lowest means were found with the Idcams and Idall implants, which are both made by the same manufacturer (IDI^®^), with Sku = 3.1 (2.9–3.2) (Table 2). This parameter is highly significant, as it provides qualitative information on roughness. Besides this, however, the nature of the peak slopes and spacing of irregularities undoubtedly have a determinant role on the adherence of bone cells.

C. Ssk

The Ssk parameter represents the skewness or degree of symmetry of the surface heights. The averages of three measurements taken on each implant were positive, indicating that the roughness of all analyzed implants was primarily textured, not flat. The mean Ssk for all samples was 0.4 (0.1–0.8) (Table 2).

D. Sz

This parameter represents surface homogeneity. The higher the Sz value, the greater the slope of the analyzed surface. The mean Sz for our 13 implants was 32.6 µm (18–57.1). The Universal implant presented the highest value at 57.1 µm (49.7–64.6) (Table 2).

E. Analysis of a hybrid parameter: Sdr

This parameter represents the degree by which a surface area is increased compared to a reference planar surface, enabling assessment of roughness. The mean Sdr for all the implants we studied was 4896.1%, revealing that the surface area was increased by an average of 48.9 fold. As was the case for Sa values, the highest Sdr value was found in the SLA-active implant, at 19979.2% (13,781.7–24,856.4), indicating a 199-fold increase in surface area (Table 2). The Axone implant presented the lowest Sdr value, at 529.3 (557.8- 630.1), i.e., a 5-fold surface area increase. The results per group revealed a mean Sdr of 2021% (1856.6–2147) for A, 9313% (4463.1–18,988.7) for B, and 6166.5% (4463.1–17,990.2) for C. As was the case with Sa, we found the highest Sdr in Group B, involving implants subjected solely to sandblasting.3.1. Microstructural characterization.

The microstructure of the Osseotite dental implant^®^ is characterized by the presence of a α-phase crystals partially decorated with β-phase structures. Chemical analysis using energy-dispersive spectroscopy revealed partitioning between the aluminum and vanadium. As expected, the aluminum was found to be an alphagenic element while the vanadium was betagenic.

Global analysis of the target area also enabled us to confirm the material as a TA6V titanium alloy. To quantify the amount of β phase, the sample was assessed without chemical attack. Only the difference in atomic number enabled us to distinguish phase α from phase β. The surface fraction of β phase obtained using imaging analysis was in the region of 4%. We then compared this level to that calculated using the ThermoCalc thermodynamic code and the Saunders database. We considered a nominal composition with an oxygen concentration corresponding to extra-low interstitial levels (1200 ppm). The value calculated for thermodynamic balance was similar to that obtained in experiments, supporting the fact that TA6V titanium alloy is of ELI quality and that the alloy had been cooled sufficiently to be near balanced. The other two implants (Osseospeed^®^ and SLA active^®^) contained no β phase, suggesting that these two alloys are of “pure” composition. Chemical analysis confirmed titanium to be the majority concentration. Traces of oxygen were also detected. The most notable difference between the two microstructures consisted of the shape of the α grains. The SLA active^®^ featured significantly elongated grains, while the Osseotite ^®^ grains were equiaxed. We also noted the presence of significant porosity in the Osseospeed dental implant^®^ This porosity and equiaxed α grain shape indicates a metallurgical process of the powders was involved. This type of manufacturing approach, it should not be forgotten, has been in use by Straumann for many years now. Similarly to before, the shape of the grains can have a major impact on the material’s mechanical properties. This equiaxed shape has in the past been highly sought after as a means to significantly limit elastic anisotropy, yet the extensive presence of porosity associated with such forms weakens the material under external mechanical strain.

Furthermore, we made observations of the exterior of all three different dental implants, noting that only the SLA-active^®^ implant featured a modified microstructure on its surface with needle-shaped α phase crystallization. At this point, it should be noted that this implant had undergone sandblasting with corundum beads.

### 3.2. Surface Composition and Morphology

Based on the EDS analysis results (Table 3), the following four of the brands studied used pure Grade 4 titanium alloy in their implants: Straumann, Tekka, Euroteknika, and Biomet 3i. Anthogyr uses a Grade 5 titanium alloy or Ti-6Al-4V, while IDI Système opted for biphasic Grade 4 and 5 titanium alloy. The differences in the alloy used influence how the chemical and physical properties of the implants differ, since Grade 5 titanium alloy offers low elasticity modulus and higher resistance to corrosion than pure titanium. The physical properties of the alloy, however, are also affected by which thermal processes and thermomechanical conditions are involved in its manufacturing.

In terms of surface treatment, five of the brands analyzed used sandblasting followed by acid etching, namely: Straumann, Tekka, Euroteknika, Anthogyr, and IDI système.

This type of surface treatment creates macro-roughness from the sandblasting and micro-roughness from the etching. Yet sandblasting materials differ from one manufacturer to another, thus creating different sizes of macro-roughness and different surface compositions due to the incorporation of the sandblasting particles in the titanium’s surface. This alters the chemical homogeneity of the surface and can consequently impact its chemical properties.

Tekka, IDI système, and Straumann all use alumina (AL_2_O_3_) for sandblasting, which can lead to particles of this material being embedded in the surface. The Inkone implant^®^ was found to feature high quantities of aluminum and oxygen in its surface (17.36% Al and 50.14% O), indicating that the oxide layer on this implant’s surface is aluminum oxide, not titanium oxide. This thus compromises all the chemical and biological properties of the surface, being as they were based on a titanium oxide layer.

The Idcam implant^®^ presented a surface composition corresponding to Grade 4 and 5 biphasic titanium alloy. No trace of oxygen was found, however, so the titanium oxide layer is thus just an emerging layer only a few nanometers thick. These results lend the impression that this surface composition is similar to that described in the literature, yet on SEM analysis we found alumina particles embedded in the surface. This heterogeneous composition found in the implant surface calls into question the implant’s biocompatibility.

The Idmax implant^®^ had a different surface composition from that of the Idcam Implant^®^, with very high levels of oxygen and normal levels of titanium (55.08% O, 36.83% Ti). This implant’s surface is thus composed of titanium oxide, and these results, along with SEM imaging, confirm that the superficial layer of the implant is made up of titanium deposited after sandblasting and etching. This can consequently enable hydroxyapatite to coat the implant’s surface, which in turn can help accelerate osseointegration. Unfortunately, however, particles of alumina were also found on the surface, as with the Idcam implant^®^.

The SLA active ^®^ implant presented a surface composed of pure titanium with traces of oxygen. This finding confirms the presence of a thin titanium oxide layer. SEM imaging also confirmed the absence of any sandblasting residue, which is due to the size of particles used, along with the acid etching process applied involving a mixture of acids at high temperatures. These findings mean the SLA surface of the Straumann implant is superior to those of other brands that use alumina as their sandblasting material.

In order to resolve this issue, Euroteknika uses titanium dioxide (TiO_2_) to sandblast their implants, which can help avoid altering the surface composition. Their Axiom^®^ implant presented a surface of pure titanium (99.15% Ti), and the titanium oxidation thus consisted of a thin emerging layer just a few nanometers thick. Nevertheless, SEM imaging revealed TiO_2_ particles embedded in the surface, which modifies the titanium oxide layer’s homogeneity, lending it higher or lower concentrations according to the presence of sandblasting particles. This changed homogeneity can then alter the implant’s corrosion-resisting and biological properties.

Anthogyr uses BCP (biphasic calcium phosphate) as sandblasting material. Biocompatible and resorbable, this material enables bone tissue to form around the implant. The Axiom^®^ implant presented a composition corresponding to Grade 5 titanium alloy, confirming that the titanium oxide layer on the surface was just an emerging coating only a few nanometers thick. It also demonstrates that there were no calcium phosphate particles embedded in the surface, raising questions about their role in enabling bone tissue formation around implants.

Biomet 3i opted to avoid the entire sandblasting process so as to prevent experiencing any issues with particles embedding in their implants’ surfaces, thus affording them better, more precise control of the chemical composition of their implants. They instead apply double acid etching to create the desired roughness on their implants’ surfaces. This creates a clean implant surface that features a chemical composition matching that of the titanium alloy used. One example of this type of surface is OSSEOTITE^®^, composed of pure titanium with a thin layer of titanium oxide.

The NANOTITE^®^ implant, according to its manufacturers, features an OSSEOTITE surface coated with a layer of calcium phosphate aimed at reducing bone tissue formation around the implant. Our analysis of this surface revealed a presence of calcium (1.40% Ca) that we deem too low to have any effect on bone formation around the implant. Further, more extensive studies are now required to determine the thickness of the coating on this surface and speed of resorption in order to identify the advantages and drawbacks of such implants.

- Surface morphology

SEM imaging enabled analysis of the size of macro- and micro-roughness on the surface of each implant, as well as the homogeneity or heterogeneity of their roughness.

The five analyzed implants, each of which had been subjected to sandblasting followed by acid etching, presented surface roughness varying from 2 to 20 µm, in line with the literature.

**Figure 1:**
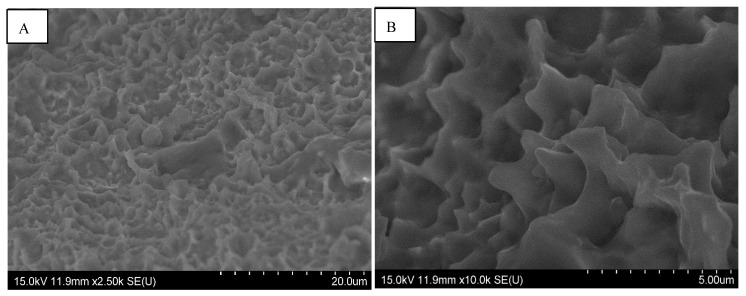
SEM image of the surface morphology of the Straumann^®^ implant showing a very regular surface area and an oriented surface roughness: magnification ×2500 (**A**) and ×10,000 (**B**).

**Figure 2:**
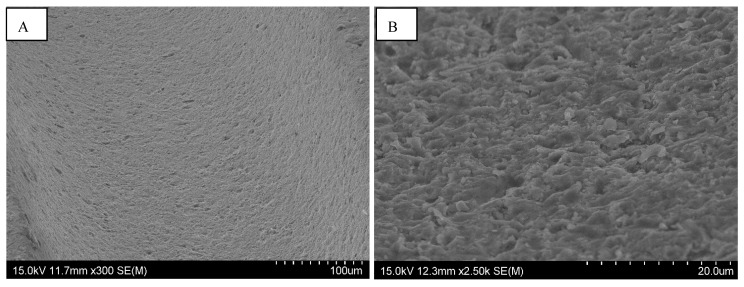
SEM image of the surface morphology of the Tekka Inkone^®^ implant showing a regular surface: magnification ×300 (**A**) and ×2500 (**B**).

**Figure 3:**
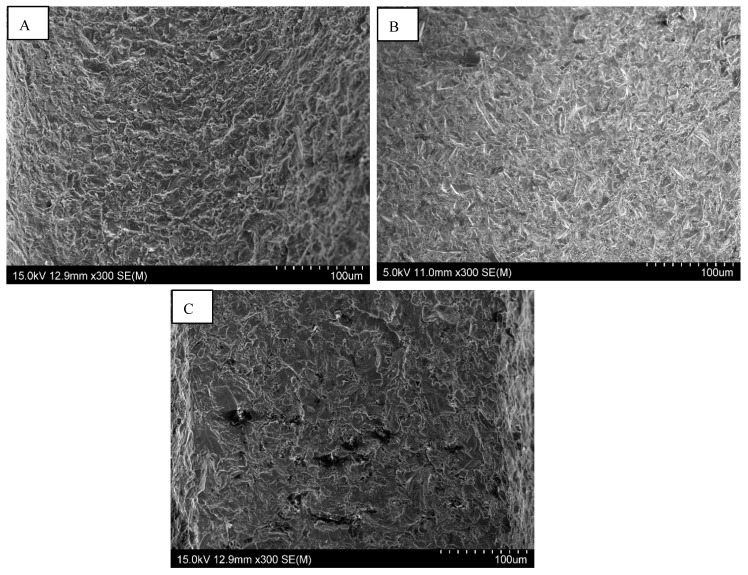
SEM images of the Axiom^®^ implant surface morphology from Anthogyr (**A**); Natea^®^ from Euroteknika (**B**) and Idcam^®^ IDI System (**C**) showing irengular surfaces: magnification ×300.

The difference we observed between them concerned the regularity of the roughness. The Straumann and Tekka Inkone implants presented regular surfaces (Figure 1 and Figure 2), while the Anthogyr, Etroteknika, and IDI Système implants had more irregular surfaces (Figure 3). This difference in morphology is due to variation in the size of particles used in the different sandblasting processes.

**Figure 4:**
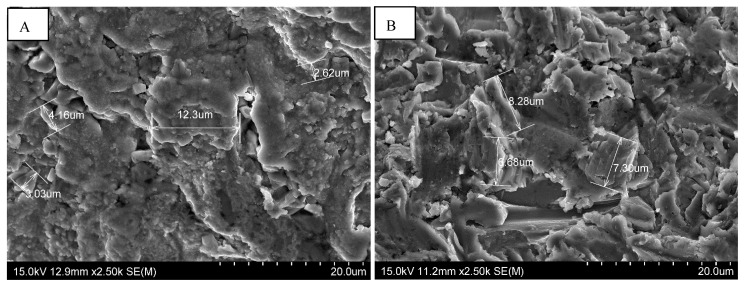
SEM images of Idcam^®^ implants from IDI System (**A**) and Natéa^®^ from Euroteknika (**B**) showing a Nanomeric appearance of surfaces: Magnification ×2500.

At very high magnification, nanometric-sized surface roughness was observed on the Euroteknika and IDI Système implants (Figure 4). This quality of nanometric surface roughness is supposed to significantly accelerate osseointegration around the implant, while also increasing its biomechanical resistance.

**Figure 5:**
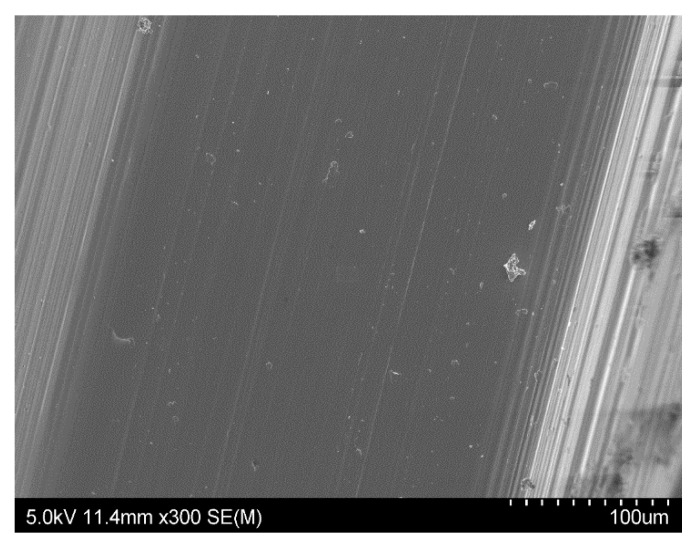
SEM image of a rough machined surface of the Biomet 3i OSSEOTITE^®^ implant: Magnification ×300.

The surfaces of the OSSEOTITE^®^ and NANOTITE^®^ implants presented no significant difference regarding morphology, and only differed in terms of their micro-roughness of 2–5 microns in size, which is to be expected for materials that have not undergone sandblasting. Imaging revealed no significant difference in surface morphology following the formation of a layer of calcium phosphate. One of the OSSEOTITE^®^ implants, however, presented a completely smooth surface (raw factory quality) (Figure 5) that had undergone no surface processing at all, which calls into question the quality control procedures in place at Biomet 3i and other manufacturers.

(a) Straumann

Analysis of the Straumann implant’s surface confirmed the use of pure titanium for their implants. The quantitative results indicate that the composition is 100% titanium (Table 4).

**Figure 6:**
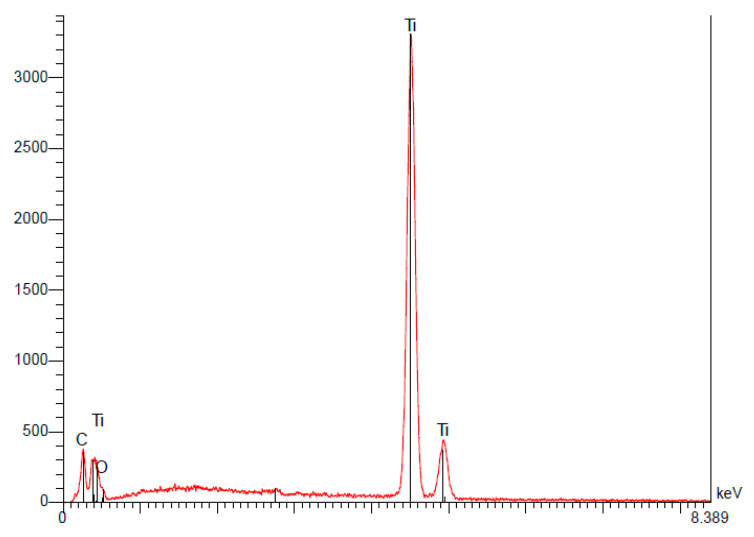
Spectrum of the Straumann implant.

Traces of oxygen were also detected on the surface, confirming the presence of a thin layer of oxides (Figure 6).

**Figure 7:**
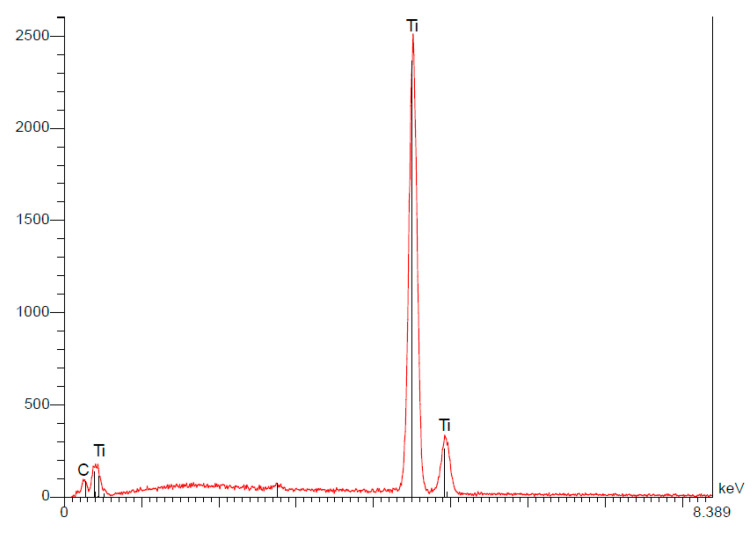
The spectroscopy of the Straumann implant’s neck.

To confirm the presence of a thin layer of oxides, we also analyzed the smooth neck of the implant (smooth surface not subjected to any SLA processing). The results demonstrate there was no oxygen in this area (Figure 7) (Table 5).

(b) Tekka Inkone^®^

Analysis of this implant’s surface revealed the presence of 32.50% titanium, 17.36% aluminum, and 50.14% oxygen, in concentrations by weight (Table 6).

**Figure 8:**
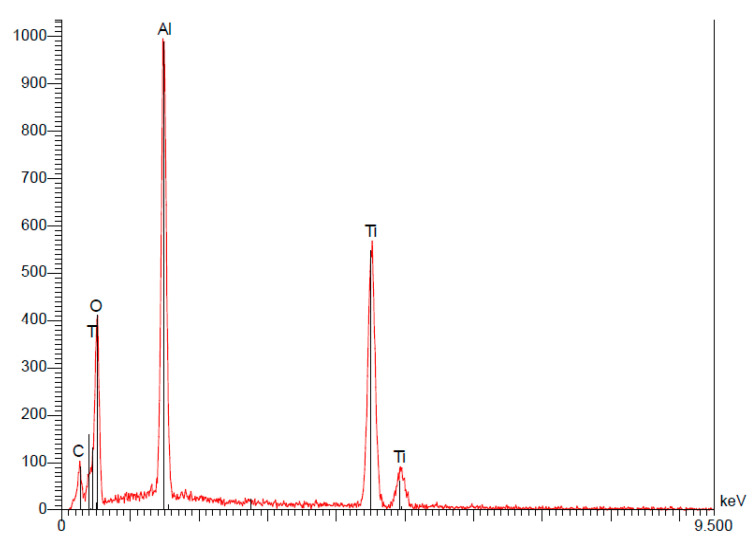
Spectroscopy of the Ikone^®^ implant.

These results confirmed the presence of oxidation on the surface, yet the high levels of oxygen and aluminum were due to the presence of Al_2_O_3_ following the sandblasting (Figure 8).

**Figure 9:**
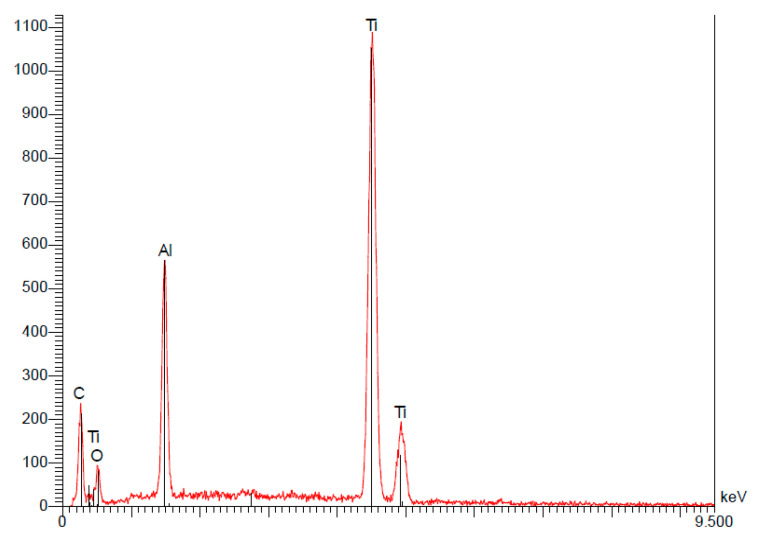
The spectroscopy of the Ikone implant’s neck.

At the neck of the Inkone implant, we found a composition of 67.29% titanium, 10.92% aluminum, and 21.79% oxygen, in weight percentages (Table 7). These results are explained by the implant’s neck having undergone the same surface treatment as the rest, although the residual concentration of aluminum is lower at the neck (Figure 9).

## 4. Discussion

How an implant initially reacts to the bone tissue of the implanted patient is affected by the bodily fluids that come into direct contact with its surface. A layer of macromolecules and water covers the implant surface, following which a series of interactions occur between these cells and the surface, leading to the release of certain chemotactic factors and growth factors, which then affect the cellular activity in the tissue surrounding the implant. This leads us to believe that the difference in cell behavior occurring around the implant is directly related to the composition and topography of its surface.

On the other hand, surface roughness has an effect on the proliferation, differentiation, and synthesis of proteins (including growth and regulation factors). Furthermore, this lends the implant better wettability, and thus better bone-implant contact (BIC).

In our study, six different dental implants from different brands were compared. The comparison focused solely on composition, however, via EDS analysis of the surface, as well as surface morphology using SEM.

(a) Composition

The chemical composition of an implant’s surface is influenced by what type of titanium alloy is used in its manufacturing, as well as the different chemical and physical treatment processes that are applied to its surface. Titanium has been shown to offer excellent chemical inertia, resistance to corrosion, and biocompatibility, due to its chemical stability and the titanium oxidation layer that forms on its surface. Any alteration of this oxide layer could thus alter these chemical properties.

Analysis of the implant’s surface composition enables us to confirm how well-adapted the titanium alloy chosen by each manufacturing is, as well as to determine how their chemical composition was affected by the various surface treatments.

Grade 5 titanium alloy (also called TA6V, Ti 6Al 4V, Ti6-4; i.e., titanium aluminum vanadium alloy, composed of: O < 0.20; Fe < 0.40; H < 0.015; C < 0.10; N < 0.05/Al = 5.5–6.75/V = 3.5–4.5), is a biphasic alloy, with an alpha-beta crystalline structure. The presence of aluminum, an alphagenic element, lends the alloy better mechanical resistance. The vanadium, a betagenic element, further increases mechanical resistance while also boosting corrosion resistance. In addition, clinical studies analyzing this alloy have reported there to be no contra-indications for its use, which is already widespread.

(b) Titanium reactivity

It is a mistake to believe that titanium is perfectly neutral and stable. As with all metals, it becomes oxidized in the mouth in the presence of other metals, releasing ions (metallic particles), which spread into neighboring tissues and then around the body. Furthermore, titanium alloys contain aluminum (also released by ionization), which is a neurotoxin known to play a role in Alzheimer’s disease and which competes with calcium in the formation of hydroxyapatite.

Oral electro-galvanism is known to lead to degenerative conditions affecting the nervous system. These electrical currents, which can reach intensities of several hundreds of microvolts, hamper nerve cell function. Given the electrical nature of the disturbances they cause, it can thus encourage neurodegenerative conditions to develop, such as Alzheimer’s disease or even multiple sclerosis, which has been posited as being caused by slow electrocution of the cells [16].

During the stage of osseointegration of an endosseous implant, a vast majority of cases are affected by bone loss, thus causing craterization of the implant. This, in turn, leads to the implant’s surface being exposed at the gum and then causing oral electro-galvanism. Hence the importance of insulating this surface, since it could become exposed. It is advisable to keep in mind the kinetic processes of the treatments provided. It is impossible, for example, to design a combination of all possible restorations and processes of just one patient in order to create the perfect balance. Each patient’s medical history comprises a series of actions and decisions that accumulate and combine to lead to pathogenic electro-galvanism. This is why it is the most recently developed medical elements that must adapt to the patient’s mouth and not the patient that must undergo interventions to adapt our materials.

Grade 4 titanium materials that are not alloys are naturally the most commonly used as they offer the best compromise between the properties of mechanical resistance, ductility, and corrosion resistance. Nevertheless, the compositions of implants currently on the market differ widely, and there does not appear to be a set standard.

Surfaces used in the past were more or less smooth on leaving the factory. The Sa of these machined surfaces varies from 0.53 µm and 0.84 µm depending on what manufacturing conditions were applied. Furthermore, the bone reaction to these surfaces is of the corticalization type.

The manufacturing process has since evolved, creating implants with rough surfaces with physical and chemical subtractive processes. These two processes lead to bone tissue response of a trabeculation type.

Nevertheless, the machined-surface implants used by Branemark [1], have now been in use for 30 years. This provides us with enough hindsight to prove the merit of this line, and nothing can in any way deny that these manufactured implants have proven their value. Lekholm et al. [17] notably reported a success rate of 93.3% for implants in partially edentulous patients, with an accumulated mean bone resorption of 0.6 mm for a follow-up of 5 years. A similar study by Henry et al. [18] on single-tooth replacements reported a success rate of 100% for mandibular implants and 96.6% for maxillary ones, with minimal resorption of the marginal bone. Lindquist et al. [19] published that of the 273 implants supporting fixed mandibular prostheses they observed, marginal bone resorption was 0.9 mm after 10 years and 1.2 mm after 15, with only three implants lost.

Despite these successes, implant surfaces have had to rapidly evolve to fulfil the growing demand for faster healing. Factory-treated osseointegrated implants seem to have long-term success, yet short implants inserted in posterior positions with low-density bone are less successful. Furthermore, clinical literature has shown that resorption of crestal bone around implants tends to be inhibited by the screw threads or porous surface texture. In another study, Quirynen et al. [20] reported greater marginal bone loss around the machined sections compared to the screw threads. Comparable extents of exposure were also published by Pilliar et al. [21].

Hence the modification of implant surfaces using a TPS (Titanium Plasma Spray) or hydroxyapatite coating, as well as the more recent approach of creating roughness on commercial-grade pure titanium using sandblasting or acid etching, which seem to improve rates of osseointegration. A past experiment analyzing the number of osteoblastic cells adhering to titanium disks with varying contours reported these cells attaching in significant numbers to surfaces roughened via sandblasting with 50 μm grains [22].

In 1991, Buser et al. [23] demonstrated that increased surface roughness correlates with increased bone contact, in percentage, around the implant. It’s known that rough implant surfaces offer greater direct bone apposition, while machined implants result in varying degrees of fibrous encapsulation. A study, by Rompen et al. [24] used resonance frequency and reported that between 0 and 6 weeks of healing there was a significant decrease in implant stability quotient in machined implants, but not for those coated with oxidation following anodizing. These results demonstrate that rough “oxidized” implants offer stronger bone-implant contact than machined ones, doubtless due to the faster and stronger attachment they enable. Larsson et al. [25] in 1996 came to similar conclusions, as did Thomas and Cook [26] in 1987 with other types of rough surfaces. Klokkevold et al. [27], on the other hand, reported mean removal torque values with the medium-rough Osseotite^®^ surface that were four times those with machined surfaces.

In 1991, Burchard et al. [28] concluded that cells attach better, one way or another, on rough surfaces than on machined ones. Results to this effect were published in an in vitro study suggesting that cells adhere better to sandblasted surfaces compared to machined ones [29]. It has also frequently been observed that the surface density of proteins or cells is much higher on rough or porous surfaces. Cochran et al. [30] however, observed periodontal cells attaching and proliferating on both machined and rough surfaces. They found that this type of cell tended to attach more easily to machined surfaces, thus confirming the need for a machined neck.

It has been proven that, once cells are in contact with an implant surface, the phenotypical expression of osteoblasts is affected by the topography and topology of the titanium surface. Notably, Martin et al. [31] demonstrated in their 1995 study that MG63 cells, similar to osteoblasts, growing on disks of titanium of different degrees of roughness, presented different responses in terms of cellular morphology, cell proliferation, phosphatase-alkaline activity, RNA synthesis, and protein production. In general, the number of cells found on the roughest surfaces was smaller, and the proliferation rate lower, yet matrix formation was increased compared to that observed with machined surfaces. This only confirms the results of Groessner-Schreiber and Tuan [32], who showed that the roughest titanium surfaces increased phosphatase-alkaline activity and calcification in cultures of chick embryo osteoblasts.

Further to these findings, another study by Kieswetter et al. [33] cited in this work, demonstrated that roughness has an impact on another type of osteoblast production, that of prostaglandin E2 (PGE2) and growth factor TGFβ1, both of which are involved in bone healing and growth. They observed that as surface roughness increased, the synthesis of these factors was boosted. The increased production of these local factors by cells of rough surfaces indicates that such surface cells are more heterogeneous and thus more suited to synthesis than machined surfaces. Despite smaller numbers of cells being found proliferating on these rough surfaces, the total levels of PGE2 and TGFβ1 are greater and, thus, so is the cellular activity. Hence, the impact of surface roughness, in a purely mechanical way, on the production of autocrine/paracrine factors by cells at the interface between bone and biomaterial, which could then dictate what type of interface is created at the implant site.

Surface roughness can also alter cellular response to extrinsic regulatory agents, such as $1alpha,25-{\left(OH\right)}^{2}D^{3}$ (vit. 1alpha). In their study discussed above, Boyan et al. [34] demonstrate that, in normal conditions, MG63 cells respond to vit. 1alpha by decreasing cell proliferation and increasing phosphatase-alkaline activity and production of osteocalcin, LTGFβ, and PGE2. Yet the surfaces with the highest roughness presented significantly greater responses to the vitamin D metabolite. This is potentially due to the cells growing on this type of surface presenting a more varied phenotype than normal. Several studies have, in fact, reported the effect of 1alpha on osteoblasts to be directly linked to the differentiated state of bone cells [35,36].

While it has been clearly proven that an active surface speeds up and increases the rate of osseointegration, the effects on immediate loading have rarely been studied. Still, preliminary studies in animals show promising results. Short-term clinical trials have demonstrated rough surfaces having positive effects on early loading after just two months of healing. These have shown that surface roughness boosts the mechanical interfacing between the implant surface’s and bone’s macromolecules, leading to increased resistance to compression, tension, and shearing forces [26,37]. Furthermore, Gotfredsenet et al. [38] published evidence that sandblasted titanium implants require up to three times the removal torque to extract them compared to machined implants.

Some authors have also suggested that textured surfaces provide the type of mechanical link needed between metal and bone to ensure adequate stimulation of bone tissue [26]. Rather than being due to some sort of mechanical interlocking structure, this is due to the screw threads, grooves, pit, and notches creating a heterogenic field of force vectors within the surrounding bone during muscular activity. Thus, a low “magnitude” of stress balances out the strain required to create the specific bone cell alignment needed to stimulate the synthesis of new bone.

In 1990, Wilke et al. [39] manufactured screw-based implants featuring six different surface structures, measuring the removal torque at the point of implant extraction. They found that surface roughness and the chemical processes applied to the surface had a significant impact on the shearing force that had to be applied (higher for rough surfaces) to the interface location. The rough or active surfaces were capable of increasing the percentage of osseointegration. Studies performed on low-density human bone have also demonstrated that modifying the surface of implants can increase the rate of bone-implant contact yet cannot impact on bone density surrounding the implant during healing. The next step is to determine whether a surface can be modified so as to improve implant survival over the long term in the most critical implant sites.

Nevertheless, Murray et al. [40] previously reported, in 1989, that rough surfaces influence bone resorption around the implant, increasing it by two-fold compared to machined surfaces. Since then, however, it has been demonstrated that overly rough surfaces can have damaging effects, such as extremely significant strain poorly distributed around the implant. Orthopedic studies have actually demonstrated that the bone reacts differently depending on what strain and stress conditions are applied. Frost’s “mechanostat” theory outlines four different load thresholds: low strain level, the standard level of strain capable of maintaining constant bone mass and density, a slightly higher strain level capable of inducing positive remodeling and bone hypertrophy, and the strain overload level causing bone resorption [41]. The load capable of causing bone resorption appears to be dependent on the occlusal forces in play, and this could be why peri-implant issues occur after initial loading. Yet a constant load is capable of causing bone hypertrophy, which increases the bone’s ability to resist a load strain. When the right balance is struck, the implant survives, but if the overload threshold is exceeded all around the implant, it progressively loses its level of osseointegration. The conditions surrounding the bone-implant interface have a crucial effect on the model of bone load around the implant. Not only does this impact load level, but the distribution of stress and strain in the bone is significantly influenced by the interface conditions, as this is directly linked to the implant’s surface condition.

As described above, several methods are now available today to obtain surface roughness. We can thus create different “morphologies” of roughness depending on which technique is used. This enables us to draw comparisons between different roughness profiles.

In addition, several other studies have demonstrated that a titanium implant subjected to sandblasting then dipped in acid (SLA implant^®^) offers better bone-implant contact over the short term than one with a surface of titanium sprayed with plasma (TPS implant^®^), in “non-oral” bone. One particular study, by Cochran et al. [42] compared x-ray imaging of SLA implants^®^ to that of TPS implants^®^ over 15 months. They analyzed 69 implants inserted in dog jaws, performing the first X-ray on the implantation of the prosthesis (evaluating the integration period), then again at three months, six months, nine months, and 12 months of load-bearing. DIB measurements revealed that the SLA implants presented significantly less vertical bone loss (0.52 mm) than the TPS implants (0.7 mm), both before the implant was subjected to any strain and after three months of load-bearing (0.73 mm versus 1.06 mm). This difference was maintained throughout the first year of follow-up. The same trend was observed for CADIA measurements, where the SLA implants^®^ presented greater crestal bone density.

As for the Martin et al. [29] study, they demonstrated that the phosphatase-alkaline activity of the osteoblasts was more significant on SLA implant^®^ surfaces than on those of TPS implants^®^, signifying greater cellular differentiation. First and foremost, these results demonstrate that both types of implants achieve and maintain highly successful tissue integration. Then again, the SLA surface produced better results than that of the TPS implants^®^ during the first two years. This is in line with the findings of Buser et al. [43] in their study on removal torque. An overly rough surface (TPS) prevents cells or large molecules to penetrate through surface crevices in certain areas due to the peaks being grouped too close together.

## 5. Conclusions

The various processes used by manufacturers today offer us a range of different surfaces to choose from that vary widely both in terms of topography and degree of roughness. Some hinder bone colonization, thus limiting osseointegration, while others encourage these cells to attach, thus ensuring effective integration.

In terms of all the different types of surfaces, it is near impossible to define one as more satisfactory than another. Such a determination would require first conducting long-term research into all implants, with well-defined, identical objectives and criteria for success. Such studies would seek to evaluate, over many years, the real risks posed by different surface roughness qualities, such as ion or particle release.

In any case, with the studies available to us today, it seems clear that rough surfaces do not provide poorer results than machined surfaces in the first year of use, though the latter is most often found to be superior. Furthermore, future perspectives for rough surfaces could be to consider them as supports for molecules to stimulate cell differentiation, proliferation, and synthesis. It is in this capacity that surfaces such as TiUnite or SLA, which have already proven able to ensure very effective osseointegration, offer great possibilities given the potential we have of controlling their manufacturing parameters and thus their roughness and porosity conditions.

The TPS surface, on the other hand, presents a surface that is too rough, limiting cell adhesion and proliferation while also causing the release of too many titanium particles which hinders osseointegration. This type of coating also appears to have fallen out of favor among practitioners, with Nobel Biocare and Straumann notably stopping their use of the system.

Regardless, further studies are still needed to define the optimal surface that is sufficiently rough, yet not too rough, and sufficiently bioactive to obtain the best osseointegration.

In the study we present herein, we have demonstrated the different processes available for modifying titanium surfaces with the aim of improving the osseointegration of titanium implants, as well as the different surfaces that are currently available on the market. The development of these surfaces has been empirically achieved via many in vitro and in vivo experiments. The majority of these experiments were not standardized and used different surfaces, cell populations, and animal models. Furthermore, the precise role played by chemical agents and surface topography on early osseointegration of titanium implants has yet to be properly studied. In addition, there have been hardly any comparative studies on different implant surfaces performed.

Our comparison of the surface conditions and compositions of different implants on the market today has enabled us to reveal the differences between these implants, as well as underlining the absence of any independent recognized standard for the condition of dental implant surfaces. We also highlight the absence of any independent quality control process of the manufacturers’ processes.

The future of dental implantology should look to developing surfaces with controlled and standardized topography and chemical composition. By doing this, we will be able to better understand how the body’s tissues and cells interact with implant surfaces. Using materials that stimulate osseointegration in the peri-implant space could also offer a solution in difficult clinical cases when bone density and quality is poor. It is our belief that, in time, these treatment strategies will improve the process of osseointegration of dental implants, ensuring both their immediate effectiveness and long-term success.

## Figures and Tables

**Table 1:** Names, manufacturers, dimensions, manufacturing processes, and classifications of all 13 implants analyzed.

Group	Implants	Manufacturer/Country	Size: Diameter and Length	Surface	Classification
Group A	Osseotite^®^	Biomet 3i, Palm Beach Gardens, FL, USA	4 by 13	Double acid etching	Subtractive
Group B	Leone Implant^®^	Leone, Trezzano, Italy	4.1 by 12	Sandblasting	Subtractive
	EVL^®^	Serf, Décines-Charpieu, France	4 by 14	Sandblasting with high-purity alumina particles	Subtractive
Group C	Idall^®^	Implant Dentaire International, Montreuil, France	4 by 11	Sandblasting and acid etching	Subtractive
	Idcams^®^	Implant Dentaire International, Montreuil, France	4 by 11	Sandblasting and acid etching	Subtractive
	Ossespeed^®^	Dentsply Sirona Implants, Mannheim, Germany	4 by 17	Titanium bead sandblasting and acid etching	Subtractive
	SLA Active^®^	Straumann, Fontenay-sous-Bois, France	4.1 by 12	Corundum Sandblasting and HCl/H_2_SO_4_ acid etching	Subtractive
	SLA^®^	Straumann, Fontenay-sous-Bois, France	4.1 by 12	Corundum Sandblasting and HCl/ H_2_SO_4_ acid etching	Subtractive
	Natea^®^	Euroteknika, Sallanches, France	4.8 by 14	Titanium oxide bead sandblasting and hydrofluoric acid etching	Subtractive
	Universal^®^	Euroteknika, Sallanches, France	4.8 by 14	Titanium oxide bead sandblasting and hydrofluoric acid	Subtractive
	Natural^®^	Euroteknika, Sallanches, France	4.8 by 14	Titanium oxide bead sandblasting and hydrofluoric acid	Subtractive
	In-Kone-Universal^®^	Tekka, Lyon, France	4.5 by 10	Corundum Sandblasting and acid etching	Subtractive
	Axone^®^	Anthogyr, Sallanches, France	4 by 11	BCP sandblasting and acid etching	Subtractive

**Table 2:** Results of the roughness parameters analyzed [15].

Group	Implants	Sa (µm)	Sq (µm)	Ssk	Sku	Sv (µm)	Sz (µm)	Sdr (%)
Group A	Osseotite^®^	2.2 (2.1–2.4)	3.2 (3.2–3.3)	0.2 (0.2–0.3)	3.4 (3.1–3.9)	30.6 (23.7–41.8)	21.1 (20–21.8)	2021 (1856.6–2147)
Group B	LEONE implant^®^	2.7 (2.4–3.4)	3.6 (3.1–4.4)	0.2 (0.1–0.3)	4.1 (3.9–4.3)	48.4 (46.1–52)	34.8 (31.5–39.2)	5995.8 (4463.1–8988.7)
	EVL^®^	3.2 (2.9–3.7)	4.2 (3.9–4.9)	0.6 (0.5–0.7)	5 (4.5–5.5)	70.5 (57.8–78.4)	46.5 (40.54–55.39)	12,630.2 (8857.5–17,990.2)
Group C	IDALL^®^	2.1 (1.9–2.3)	2.6 (2.4–2.8)	0.3 (0.2–0.3)	3.1 (2.9–3.3)	25.5 (23.5–28.5)	19.3 (18.6–20.1)	1458.3 (1360.3–1653.8)
	IDCAMS^®^	2.2 (1.8–2.6)	2.7 (2.2–3.3)	0.3 (0.2–0.3)	3.1 (2.9–3.2)	26 (22.1–28.9)	20.6 (18.3–23)	2695.2 (2098.6–3120.1)
	Osseospeed^®^	1.8 (1.5–2.4)	2.5 (1.9–3.6)	0.8 (0.4–1.4)	8.9 (7.2–11.1)	75.9 (67.4–84.4)	33.6 (26–47)	1890.329 (443.5–4759.8)
	SLA active^®^	3.7 (3.3–4)	4.9 (4.3–5.3)	0.5 (0.4–0.5)	5 (4.7–5.4)	77.7 (64.6–87.4)	56.3 (47.4–62.2)	19,979.2 (13,781.7–24,856.4)
	SLA^®^	3.2 (2.8–4.1)	4.2 (3.6–4.7)	0.6 (0.6–0.7)	3.9 (3.7–4.1)	52.3 (48.6–55.8)	40 (33.9–45.5)	10,125.36 (6815.6–13,495.5)
	NATEA^®^	3.3 (2.2–4.7)	4.3 (2.9–6.3)	0.2 (–0.003–0.3)	4 (3.9–4.2)	49.7 (38.2–67.8)	34.41 (26.5–46.9)	3619.14 (1674–6925.1)
	UNIVERSAL^®^	2.5 (2.4–2.5)	3.2 (3.2–3.3)	0.4 (–0.06–0.6)	4.5 (4–4.8)	56.4 (49.4–62.4)	33.7 (30.6–39.1)	6687 (5853.1–8318.1)
	NATURAL^®^	3.6 (3.2–4)	4.9 (4.3–5.4)	0.6 (0.5–0.8)	5.4 (5.3–5.5)	88.4 (71.2–105.8)	57.1 (49.7–64.4)	13,456.29 (11,094.9–16,664)
	In-Kone-Universal^®^	1.7 (1.7–2.8)	2.3 (2.1–2.4)	0.1 (–0.2–0.3)	3.7 (3.2–4.7)	22.3 (19.3–26.1)	18 (16.2–20.1)	1225.51 (1042.6–1467.2)
	Axone^®^	2.3 (2.2–2.4)	3.0 (2.9–3.1)	0.4 (0.25–0.53)	5.3 (4.9–5.6)	54.6 (49.6–58.6)	32.9 (29.9–35)	595.3 (557.8–630.1)
	Mean Values	2.7 (1.7–3.7)	3.5 (2.3–4.9)	0.4 (0.1–0.8)	4.6 (2.9–11.1)	52.2 (22.3–88.4)	32.6 (18–57.1)	4896.1 (595.3–19,979.2)

**Table 3:** Presents the results regarding the composition of the implant surfaces we analyzed [15].

Implant Analyzed	Titanium Ti	Aluminium Al	Vanadium V	Oxygen O	Calcium Ca
SLA^®^	100%	-	-	-	-
Error	2.1417	-	-	-	-
Inkone^®^	32.50%	17.36%	-	50.14%	-
Error	0.8874	13.5443	-	2.8538	-
Natea ^®^	99.15%	0.85%	-	-	-
Error	1.0917	0.5505	-	-	-
Axiom^®^	89.86%	7.41%	2.73%	-	-
Error	1.1965	3.8737	0.9971	-	-
Idcam^®^	91.80%	6.99%	1.21%	-	-
Error	1.0833	1.1828	0.9028	-	-
Idmax^®^	36.83%	8.90%	-	55.08%	-
Error	1.0546	4.8059	-	6.9522	
Biomet 3i^®^ OSSEOTITE	100%	-	-	-	-
Error	1.0563	-	-	-	-
Nano Tite^®^	90.38%	-	-	7.99%	1.40%
Error	4.6713	-	-	1.4913	4.2821

**Table 4:** The quantitative results of the EDS analysis of the Straumann implant demonstrating weight percentages (P%) and atomic percentages (A%) of the elements detected [15].

Elt	Line	Int	Error	K	Kr	P%	A%	Formula	Ox%	Cat#
C	Ka	67.5	14.7604	0.0000	0.0000	0.00	0.00		0.00	0.00
0	Ka	0.0	0.0000	0.0000	0.0000	0.00	0.00		0.00	0.00
Ti	Ka	1200.8	2.1417	1.0000	1.0000	100.00	100.00		0.00	0.00
				1.0000	1.0000	100.00	100.00		0.00	0.00

**Table 5:** The quantitative results of the EDS analysis of the Straumann implant’s neck demonstrating weight percentages (P%) and atomic percentages (A%) of the elements detected [15].

Elt	Line	Int	Error	K	Kr	P%	A%	Formula	Ox%	Cat#
C	Ka	29.2	9.4161	0.0000	0.0000	0.00	0.00		0.00	0.00
Ti	Ka	1306.6	1.5635	1.0000	1.0000	100.00	100.00		0.00	0.00
				1.0000	1.0000	100.00	100.00		0.00	0.00

**Table 6:** The quantitative results of the EDS analysis of the Inkone^®^ implant demonstrating weight percentages (P%) and atomic percentages (A%) of the elements detected.

Elt	Line	Int	Error	K	Kr	P%	A%	Formula	Ox%	Cat#
C	Ka	64.5	2.8538	0.0000	0.0000	0.00	0.00		0.00	0.00
0	Ka	211.3	2.8538	0.2104	0.1046	50.14	70.33		0.00	0.00
Al	Ka	628.8	13.5443	0.2268	0.1128	17.36	14.44		0.00	0.00
Ti	Ka	615.9	0.8874	0.5628	0.2799	32.50	15.23		0.00	0.00
				1.0000	0.4974	100.00	100.00		0.00	0.00

**Table 7:** The quantitative results of the EDS analysis of the Inkone implant’s neck demonstrating the weight percentages (P%) and atomic percentages (A%) of the elements detected.

Elt	Line	Int	Error	K	Kr	P%	A%	Formula	Ox%	Cat#
C	Ka	76.0	2.7166	0.0000	0.0000	0.00	0.00		0.00	0.00
O	Ka	28.8	2.7166	0.0361	0.0261	21.79	42.94		0.00	0.00
Al	Ka	215.3	0.6561	0.0977	0.0706	10.92	12.76		0.00	0.00
Ti	Ka	753.4	0.9630	0.8661	0.6256	67.29	44.29		0.00	0.00
				1.0000	0.7223	100.00	100.00		0.00	0.00

## Data Availability

Data sharing not applicable.
